# Thermal Tracking of Sports Players

**DOI:** 10.3390/s140813679

**Published:** 2014-07-29

**Authors:** Rikke Gade, Thomas B. Moeslund

**Affiliations:** Visual Analysis of People Lab, Aalborg University, Rendsburggade 14, 9000 Aalborg, Denmark; E-Mail: tbm@create.aau.dk

**Keywords:** thermal, detection, tracking, sport

## Abstract

We present here a real-time tracking algorithm for thermal video from a sports game. Robust detection of people includes routines for handling occlusions and noise before tracking each detected person with a Kalman filter. This online tracking algorithm is compared with a state-of-the-art offline multi-target tracking algorithm. Experiments are performed on a manually annotated 2-minutes video sequence of a real soccer game. The Kalman filter shows a very promising result on this rather challenging sequence with a tracking accuracy above 70% and is superior compared with the offline tracking approach. Furthermore, the combined detection and tracking algorithm runs in real time at 33 fps, even with large image sizes of 1920 × 480 pixels.

## 1. Introduction

Traditionally, visual cameras, capturing RGB or greyscale images, have been the obvious choice of sensor in surveillance applications. However, in dark environments, this sensor has serious limitations, if capturing anything at all. This is one of the reasons that other types of sensors are now taken into consideration. One of these sensors is the thermal camera, which has recently become available for commercial and academic purposes, although originally developed for military purposes [1]. The light-independent nature of this sensor makes it highly suitable for detection and tracking of people in challenging environments. Privacy has also become a big issue, as the number of surveillance cameras have increased rapidly. For video recording in sensitive locations, thermal imaging might be a good option to cover the identity of the people observed, in some applications it might even be the only legal video modality. However, like any other sensor type, the thermal sensor has both strengths and weaknesses, which are discussed in the survey on thermal cameras and applications [1]. One way of overcoming some of these limitations is to combine different sensors in a multi-modal system [2].

The visual and thermal sensors complement each other very well. Temperature and colour information are independent, and besides adding extra information on the scene each sensor might be able to detect targets in situations where the other sensor completely fails. However, registration and fusion of the two image modalities can be challenging, since there is not necessarily any relation between brightness level in the different spectra. Generally, three types of fusion algorithms exist; fusion on pixel level, feature level, or decision level. Several proposed fusion algorithms are summarised in the survey [1].

It is clear that multi-modal detection and tracking systems have several advantages for robust performance in changing environments, which is also shown in recent papers on tracking using thermal-visible sensors [3,4]. The drawbacks of these fused systems primarily relates to the fusion part, which requires an additional fusion algorithm that might be expensive in time and computations. Furthermore, when applying a visual sensor, the possibility of identification and recognition of people exists, causing privacy issues that must be considered for each application.

A direct comparison of tracking performance in multi-modal images versus purely thermal images in different environments would be interesting, but this is out of scope for this paper. Here we choose to take another step towards privacy-preserving systems and work with thermal data only. While tracking people in RGB and greyscale images has been and is still being extensively researched [5,6], the research in tracking in thermal images is still rather limited. Therefore, in this paper we wish to explore the possibility of applying tracking algorithms in the thermal image modality.

### 1.1. Related Work

Two distinct types of thermal tracking of humans exist. One is tracking of human faces, which requires high spatial resolution and good quality images to detect and track facial features [7–9]. The other direction, which we will focus on, is tracking of whole-body individual people in surveillance-like settings. In this type of applications the spatial resolution is normally low and the appearance of people is very similar. We cannot rely on having enough unique features for distinguishing people from each other, and we must look for tracking methods using only anonymous position data.

For tracking in traditional RGB or greyscale video, the tracking-by-detection approach has recently become very popular [10–12]. The classifier is either based on a pre-trained model, e.g., a pedestrian model, or it can be a model-free tracker initialised by a single frame, learning the model online. The advantage of online learning is the ability to update the classifier, as the target may change appearance over time. In order to apply this approach for multi-target tracking, the targets should be distinguishable from each other. This is a general problem in thermal images, the appearance information is very sparse, as no colour, texture, *etc.*, are sensed by the camera.

Other approaches focus on constructing trajectories from “anonymous” position detections. Both online (recursive) and offline (batch optimisation) approaches has proven to be successful. Online approaches cover the popular Kalman filter [13] and particle filters [14,15]. The methods are recursive, processing each frame as soon as it is obtained, and assigning the detection to a trajectory. Offline methods often focus on reconstructing the trajectories by optimising an objective function. Examples are presented in [16] by posing the problem as an integer linear program and solving it by LP-relaxation, or in [17] solving it with the k-shortest path algorithm.

Tracking in thermal video has often been applied in real-time applications for pedestrian tracking or people tracking for robot-based systems. Fast online approaches have therefore been preferred, such as the particle filter [18,19] and the Kalman filter [20,21].

While most works on tracking people in thermal images have focused on pedestrians with low velocity and highly predictable motion, we apply tracking to real sports video, captured in a public sports arena. It is highly desired to track sports players in order to analyse the activities and performance of both teams and individuals, as well as provide statistics for both internal and commercial use. However, sports video is particularly challenging due to a high degree of physical interaction, as well as abrupt and erratic motion.

**Figure 1.**
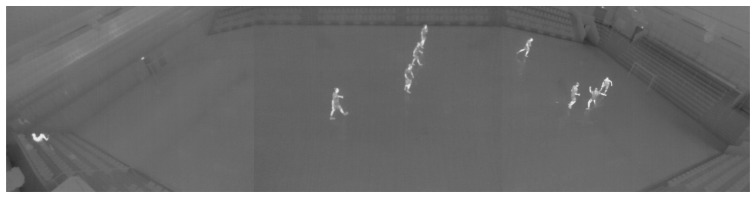
Example of a frame from the thermal sports video.

Figure 1 shows an example frame from the video used for testing. The video is captured with three cameras in order to cover the entire field of 20 m × 40 m. The images are rectified and stitched per frame to images of 1920 × 480 pixels.

This paper will investigate the applicability and performance of two different tracking approaches on thermal data. First, we design an algorithm based on the Kalman filter. Then, we test a publicly available state-of-the-art multi-target tracking algorithm [22]. The algorithms are evaluated on a 2 min manually annotated dataset from an indoor soccer game.

## 2. Detection

**Figure 2.**
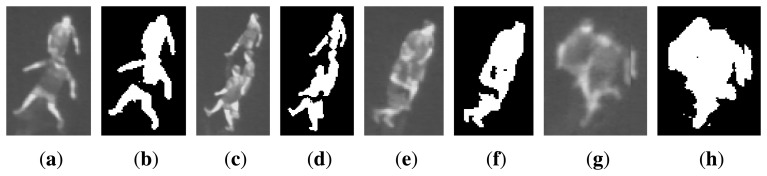
Examples of occlusions between people. For each example the corresponding binarised image is shown, found by automatic thresholding.

Detecting people in thermal images may seem simple, due to an often higher temperature of people compared with the surroundings. In this work we focus on indoor environments, more specifically a sports arena. This scene is quite simple in terms of a plain background with relatively stable temperature. Hence, people can often be segmented from the background by only thresholding the image. The challenges occur in the process of converting the binary foreground objects into individual people. In the ideal cases each blob is simply considered as one person. However, when people interact with each other, they overlap in the image and cause occlusions, resulting in blobs containing more than one person. The appearance of people in thermal images is most often as simple as grey blobs, making it impossible to robustly find the outline of individual people in overlaps. Figure 2 shows four examples of occlusions and the corresponding binarised images.

While full or severe occlusions (like Figure 2e) cannot be solved by detection on frame basis, we aim to solve situations where people are only partly occluded and can be split into single person. Likewise, we want to detect only one person even when it has been split into several blobs during thresholding. We implement three rather simple but effective routines aiming at splitting or connecting the blobs into single person. These routines are described in the following sections.

### 2.1. Split Tall Blobs

**Figure 3.**
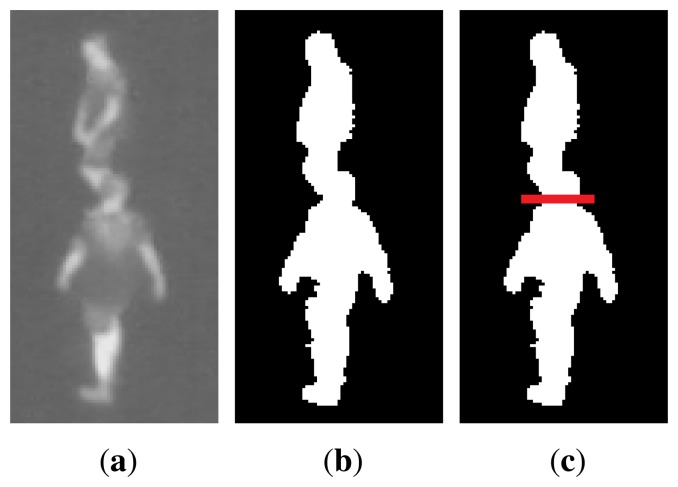
Example of how a tall blob containing two people will be split into two.

People standing behind each other, seen from the camera, might be detected as one blob containing more than one person. In order to split these blobs into single detection we here adapt the method from [23]. First, it must be detected when the blob is too tall to contain only one person. If the blob has a pixel height that corresponds to more than a maximum height at the given position, found by an initialising calibration, the algorithm should try to split the blob horizontally. The point to split from is found by analysing the convex hull and finding the convexity defects of the blob. Of all the defect points, the point with the largest depth and a given maximum absolute gradient should be selected, meaning that only defects coming from the side will be considered, discarding, e.g., a point between the legs. Figure 3 shows an example of how a tall blob containing two people will be split.

### 2.2. Split Wide Blobs

**Figure 4.**
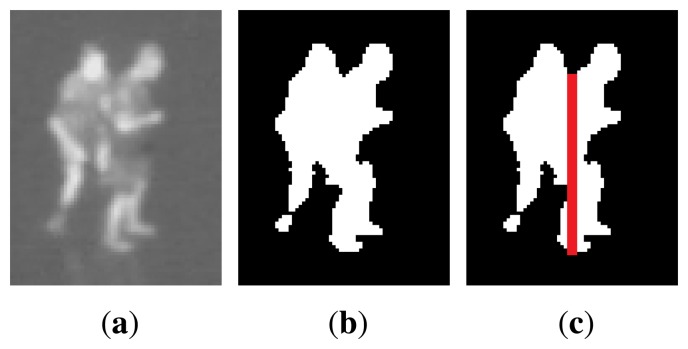
Example of how a wide blob containing two people will be split into two.

Groups of people standing next to each other might be found as one large blob. To identify which blobs contain more than one person, the height/width ratio and the perimeter are considered, as done in [23]. If the criteria are satisfied, the algorithm should try to split the blob. For this type of occlusion, it is often possible to see the head of each person, and split the blob based on the head positions. Since the head is narrower than the body, people can be separated by splitting vertically from the minimum points of the upper edge of a blob. These points can be found by analysing the convex hull and finding the convexity defects of the blob. Figure 4 shows an example of how a wide blob containing two people will be split.

### 2.3. Connect Blobs

**Figure 5.**
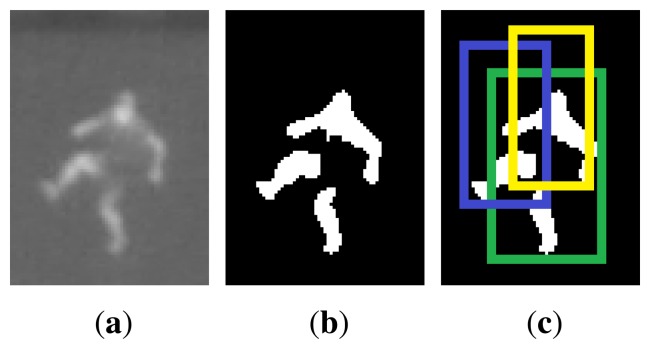
Example of a person that is split into three blobs by thresholding. Three overlapping candidates are evaluated (green, blue and yellow rectangles). Only the green candidate will be kept, because it has the highest ratio of white pixels.

One person can often be split into several blobs during thresholding if some areas of the body appear colder, e.g., due to loose or several layers of clothing. In order to merge these parts into only one detected person, we consider each binary blob a candidate, and generate a rectangle of standard height at the given position (calculated during calibration) and the width being one third of the height. For each rectangle we evaluate the ratio of foreground (white) pixels. If the ratio of white pixels is below 15%, the blob is discarded, otherwise the candidate is added for further processing. The second step is to check if the candidate rectangles overlap significantly, hence probably belonging to the same person. If two rectangles overlap by more than 45%, only the candidate with highest ratio of white pixels is kept as a true detection. These threshold values are chosen experimentally by evaluating 340 positive samples and 250 negative samples. Figure 5 illustrates this situation, where one person has been split into three blobs.

The ultimate goal for the detection algorithm is to detect each person, and nothing else, in each frame. However, with a side-view camera angle and a number of people interacting, missing detections and noise must be considered when using the detections as input for the tracking algorithms described next.

## 3. Tracking

### 3.1. Kalman Filter

The Kalman filter, introduced in the early 1960s, is a now well-known algorithm used in a wide range of signal processing applications. The recursive algorithm filters noisy measurements by predicting the next step from previous state and use the new measurement as feedback for updating the estimate. The Kalman filter estimates the state *x* of a discrete-time controlled process controlled by the linear stochastic difference equation [24]:

(1) $$x_{k}=Ax_{k-1}+Bu_{k-1}+w_{k-1}\;$$

with a measurement *z*:

(2) $$z_{k}=Hx_{k}+\nu_{k}\;$$

where *w_k_* and *v_k_* are random variables representing the process and measurement noise, respectively. The matrix *A* is the transition matrix that relates the state *x* at the previous time step *k* − 1 to the state at the current step *k*. The matrix *B* relates the control input *u_k_*_−1_ to the state *x_k_* (the control input is optional, and this term is often discarded). The matrix *H* relates the state *x_k_* to the measurement *z_k_*.

**Figure 6.**
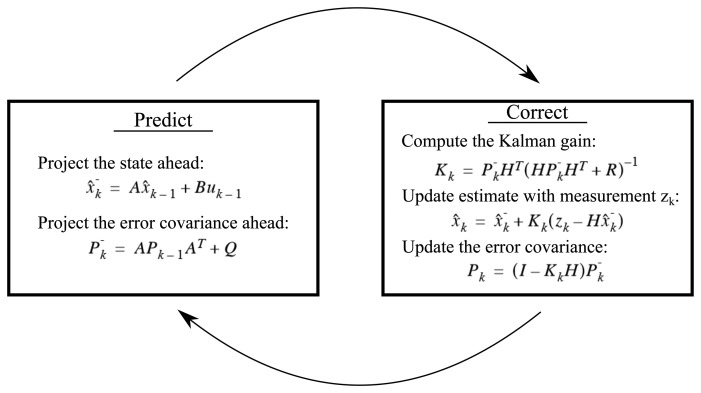
Procedure of the Kalman filter.

Figure 6 illustrates the procedure of the Kalman filter, shifting between predicting the next step from the previous state and correcting the state using a new observed measurement.

Using the Kalman filter for tracking an object in 2D, the state *x* consists of four dynamic variables; x-position, y-position, x-velocity and y-velocity. The measurement *z* represents the observed x- and y-positions for each frame.

When implementing a Kalman filter, the measurement noise covariance *R* and the process noise covariance *Q* must be tuned. *R* represents the measurement noise variance, meaning that a high value will tell the system to rely less on the measurements and vice versa.

For more details on the Kalman filter, we refer to the introduction in [24] or the original paper [13].

### 3.2. Multi-Target Data Association

Each Kalman filter maintains only the estimated state of one object. In order to keep track of several targets simultaneously, the association between detections and Kalman filters must be handled explicitly. For each frame, a list of detections are obtained as described in Section 2. Each existing Kalman filter is then assigned the nearest detection, within a given distance threshold *th*. For each detection that is not assigned to a Kalman filter, a new track is started, by creating a new Kalman filter. Kalman filters that have no assigned detections will be continued based on the predicted new positions. After a given time period without detections, experimentally set to 10 frames, the track will be terminated.

### 3.3. Tracking by Continuous Energy Minimization (CEM)

The choice of tracking based on the Kalman filter leaves no possibility for connecting broken tracks, as it is a purely recursive approach. This possibility of optimising both forward and backward in time is instead exploited in offline algorithms based on batch optimisation. We will here test one of these algorithms, using code available online. This algorithm minimises an energy function of five terms [22]:

(3) $$E(X)=E_{\text{obs}}+\alpha E_{\text{dyn}}+\beta E_{\text{exc}}+\gamma E_{\text{per}}+\delta E_{\text{reg}}\;$$

*E_obs_* represents the likelihood of object presence, determined by the object detector. *E_dyn_* is the dynamic model, using a constant velocity model. *E_exc_* is a mutual exclusion term, introducing the physical constraint that two objects cannot be present at the same space simultaneously. The target persistence term *E_per_* penalises trajectories with start or end points far from the image border. The last term, *E_reg_*, is a regularisation term that favours fewer targets and longer trajectories.

Given the set of detections for all frames, this algorithm will try to minimise the energy function (3) by growing, shrinking, splitting, merging, adding or removing until either convergence or reaching the maximum number of iterations. For further details, see [22].

The set of detections are found as described in Section 2. Being the same detection algorithm used for both Kalman filter and CEM tracker, the tracking algorithms can be compared directly.

## 4. Experiments

In this section we test the tracking algorithm on a 2-minutes (3019 frames) video from an indoor soccer game. The frames are manually annotated using bbLabeler from Piotr's Image & Video MATLAB Toolbox [25]. All frames are annotated by the same person in order to ensure consistency.

### 4.1. Kalman Tracker

The Kalman filter tracker is implemented in C# using EMGU CV wrapper for the OpenCV library [26]. Through experiments the measurement noise covariance *R* has been tuned to 0.1 and the process noise covariance *Q* is tuned to 0.002 for position and 0.003 for velocity.

### 4.2. CEM Tracker

The CEM tracker is downloaded from the author's website (http://www.milanton.de/contracking/ index.html). We use the 2D tracking option, tracking in image coordinates. Default parameter values are used, except for three parameters: Target size is reduced to ImageWidth/200 (approx. 10 pixels) due to the relatively small object size in the test video. The maximum number of global iterations is varied between 15, 30 and 60 iterations, along with the maximum number of iterations for each gradient descent, which is varied between 30, 60 and 120 iterations.

### 4.3. Results

**Figure 7.**
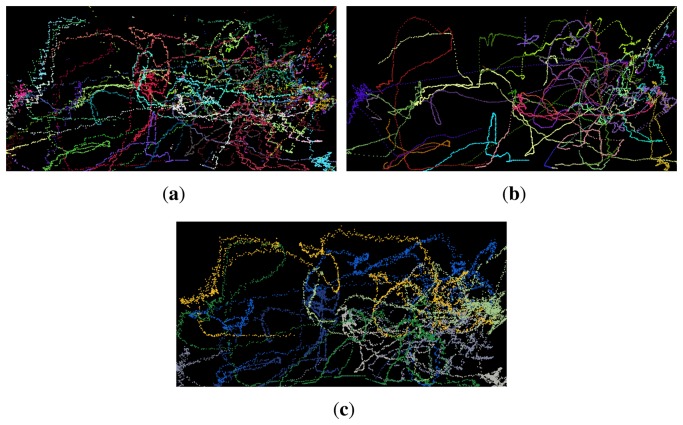
Trajectories plot in world coordinates with each identity assigned a random colour. (**a**) Trajectories found by Kalman tracker; (**b**) trajectories found by CEM tracker (60 epochs) and (**c**) manually annotated trajectories.

The trajectories found by the Kalman tracker, CEM tracker and manually annotated trajectories, respectively, are plotted in Figure 7. The trajectories are plotted in world coordinates, thus each image represents the sports field seen from above. Each new identity found by the tracker is plotted in a new colour assigned randomly. The figure shows that while the trajectories found by the CEM tracker is longer and smoother, the Kalman tracker produces more tracks, which are also very close to the ground truth.

We evaluate the tracking results using CLEAR MOT metrics [27], calculated by publicly available MATLAB code [28]. The results are measured by true positives (TP), false positives (FP), false negatives (FN), ID switches and the two combined quality measures: multiple object tracking precision (MOTP) and multiple object tracking accuracy (MOTA):

(4) $$\text{MOTP}=\frac{\sum_{i,t}d_{t}^{i}}{\sum_{t}c_{t}}\;$$

where $d_{t}^{i}$ is the distance between the object *o_i_* and its corresponding hypothesis. *c_t_* is the number of matches found for time *t*. Hence, MOTP is the total error in estimated position for matched object–hypothesis pairs over all frames, averaged by the total number of matches made.

(5) $$\text{MOTA}=1=\frac{\sum_{t}\left(FN_{t}+FP_{t}+IDS_{t}\right)}{\sum_{t}gt}$$

where *FN_t_*, *FP_t_* and *IDS_t_* are the number of false negatives, false positives and ID switches, respectively, for time *t*, while *g_t_* is the true number of objects at time *t*.

The results of the Kalman tracker and the CEM tracker with three different numbers of maximum iterations are presented in Table 1. The result for Kalman filtering is very good, considering the complexity of the data. The true positive rate exceeds 80% and the accuracy (MOTA) is 70.36%. For the CEM tracker these numbers are significantly lower, the best true positive rate is 18.14% obtained after 60 epochs. This implies a high false negative rate of 81.6%, but also a high false positive rate of 38.06%. The resulting MOTA ends up being negative. The results are clearly related to the total track length; the Kalman filter constructs more than twice the total length of tracks, which is closer to the total length of ground truth tracks of 3241.52 m.

### 4.4. Processing Time

The processing time, calculated for 3019 frames of video containing 8 people, is for the MATLAB implementation of the CEM tracker (excluding detection) with 15 epochs: 6.03 min (0.12 s per frame), 30 epochs: 8.75 min (0.17 s per frame), 60 epochs: 16.34 min (0.32 s per frame). For the C# implementation of Kalman filter tracking (with integrated detection) the processing time is only 1.55 min (0.03 s per frame).

Both methods are tested on an Intel Core i7-3770K CPU 3.5 GHz with 8 GB RAM.

## 5. Discussion

We have tested the CEM tracker with three different numbers of maximum iterations, in order to investigate whether more iterations would allow the algorithm to reach a better estimate. From 15 to 30 epochs we observe clear improvements, from a true positive rate of 11.61% to 17.07% and the false negative rate decreasing accordingly. The false positive rate increases from 27.38% to 33.19%, though. Increasing the maximum number of iterations from 30 to 60 gives only a small improvement in true positive rate from 17.07% to 18.14%, while the false positive rate increases from 33.19% to 38.06%. This indicates that further iterations will not improve the accuracy.

Given that the CEM tracker is an offline algorithm, processing a batch of frames, it is able to run the optimisation both forward and backward in time. That makes it more likely to connect broken trajectories compared with the Kalman tracker, which is recursive and needs to start a new trajectory if it loses one. As expected, this is observed as more identity switches by the Kalman tracker (219 switches) compared with the CEM tracker (51–60 switches). It is also reflected in the mean length of each trajectory; for the Kalman tracker the mean length is 11.5 m, compared with 25.2–37.2 m for the CEM tracker.

The processing time of the two algorithms indicates another big difference between online and offline approaches. The Kalman filter is well-suited for real-time applications with a processing time of only 0.03 s per frame including both detection and tracking. For the CEM tracker the detections must be saved for the full batch of frames before starting to construct trajectories. The processing time is then 0.12–0.32 s per frame, depending on the number of iterations. Furthermore, the processing time might increase significantly with the number of targets.

Both tracking algorithms are independent of the type of detection algorithm, making it possible to apply tracking to a wide range of applications. In this work we demonstrated the approach on a video from an indoor sports arena, but it could be applied directly in any scene where the human temperature is different from the background, including outdoor scenes. The performance depends on the quality of detections. In order to significantly reduce the occlusions between people, the camera could be mounted above the scene, capturing a top-view instead of the side-view shown in Figure 1.

## 6. Conclusions

We have presented an online multi-target tracking algorithm based on the Kalman filter and compared with a state-of-the-art offline multi-target tracking algorithm. In terms of accuracy the Kalman tracker is far superior in this application and constructs more than twice the total length of tracks. The drawback of this online approach is the number of split tracks and identity switches. Depending on the application and importance of identity, a post-processing method could be applied in order to optimise and connect trajectories.

## Figures and Tables

**Table 1.** Tracking results for Kalman filter and continuous energy minimization (CEM) algorithms.

	TP	FP	FN	ID Switch	MOTP	MOTA	Total Track Length	#ID's
**KF**	80.22%	9.86%	18.86%	219	0.75	70.36%	2506.78 m	218
**CEM - 15 epochs**	11.61%	27.38%	88.14%	60	0.58	−15.77%	933.66 m	37
**CEM - 30 epochs**	17.07%	33.19%	82.72%	51	0.59	−16.11%	1100.69 m	31
**CEM - 60 epochs**	18.14%	38.06%	81.60%	60	0.60	−19.91%	1228.14 m	33
